# Qu-zhuo-tong-bi decoction exerts gouty arthritis therapy by skewing macrophage polarization through butanoate metabolism

**DOI:** 10.1186/s13020-025-01162-6

**Published:** 2025-07-22

**Authors:** Siyue Song, Xianghui Wen, Fusen Chen, Jiatao Li, Kaiyue Shi, Yu Lou, Anyi Xu, Chengping Wen, Tiejuan Shao

**Affiliations:** 1 College of Basic Medical Sciences, Zhejiang Chinese Medical University, Hangzhou, 310053 China; 2 Center for Innovative, Basic Research in Autoimmune Diseases in Medicine, Hangzhou, 310053 China; 3 First Clinical Medical College, Zhejiang Chinese Medical University, Hangzhou, 310053 China

**Keywords:** Qu-zhuo-tong-bi decoction, Gouty arthritis, Gut microbiome, Butanoate metabolism, Macrophage polarization, Glycolysis

## Abstract

**Background:**

Qu-zhuo-tong-bi decoction (QZTBD), a traditional Chinese medicine (TCM), has demonstrated efficacy in the treatment of gouty arthritis. However, to date, the precise pharmacological mechanisms remain unclear.

**Purpose:**

The study aims to ascertain the therapeutic effects and the underlying mechanisms of QZTBD in the treatment of gouty arthritis.

**Methods:**

The efficacy and safety of different doses of QZTBD were investigated in *Uox*-KO mice. Candidate active ingredients were identified using UHPLC-MS/MS. The potential therapeutic pathways of the active ingredients were predicted through network pharmacology. The mechanisms of QZTBD in alleviating gouty arthritis were explored via comprehensive analyses of gut microbiota, combined with RT-qPCR, western blot, immunofluorescence, ELISA, flow cytometry, and Seahorse assay. Fecal microbiota transplantation (FMT), bacterial culture experiment, butyrate-producing bacteria (BPB) and butyrate administration, and 2-DG intervention were conducted to explore the roles of BPB and butanoate metabolism in gout progression and therapeutic mechanisms of QZTBD. In vitro studies further validated the regulatory effects of butyrate and QZTBD on macrophage polarization through glycolysis modulation.

**Results:**

18.0 g/kg/d of QZTBD effectively alleviated the symptoms of gouty arthritis with excellent hepatic and renal safety. UHPLC-MS/MS analysis and network pharmacology revealed that QZTBD exerts its effects on butanoate metabolism during gouty arthritis inflammation. QZTBD treatment increased the abundance of BPB, the levels of serum and colon butyrate, and the expression levels of *Buk* and *But*. The transplantation of QZTBD-treated microbiota reproduced the therapeutic effects of QZTBD. M1 macrophage polarization was suppressed after QZTBD intervention. The administration of BPB and butyrate attenuated gouty arthritis and orchestrated macrophage polarization. Inhibition of glycolysis regulated the phenotype of macrophage and attenuated inflammatory processes. In vitro analysis unveiled that QZTBD and butyrate modulated glycolysis to regulate macrophage polarization, thereby alleviating gouty arthritis.

**Conclusion:**

QZTBD targeted butanoate metabolism to regulate macrophage polarization, thereby effectively alleviating intestinal inflammation and restoring immune homeostasis in gouty arthritis. These findings establish a mechanistic foundation for developing precision therapeutic strategies leveraging QZTBD to combat gouty arthritis.

**Supplementary Information:**

The online version contains supplementary material available at 10.1186/s13020-025-01162-6.

## Introduction

Gouty arthritis (GA) emerges as the most common inflammatory arthritis with a global prevalence ranging from 0.68 to 3.9% among adults across diverse nations [1]. It is characterized by severe joint pain and swelling, which may progress to chronic tophaceous or erosive gout. In severe cases, this can even result in the disability or activity limitation in affected patients [2]. The acute inflammatory phase is initiated by the immune response to monosodium urate (MSU) crystals, wherein macrophages act as central mediators [3]. It is well established that macrophages exhibit a high degree of plasticity in response to environmental stimuli, and their polarization has emerged as a pivotal factor in the pathogenesis of gouty arthritis [4]. Indeed, macrophage polarization toward M1-like phenotypes mediates the initiation and progression of gout inflammation [5]. The potential efficacy of modulating macrophage polarization as a therapeutic strategy for gout has been demonstrated [6, 7].

Previous studies have shown that promoting M2 macrophage polarization alleviates hyperuricemic nephropathy in *Uox*-KO mice, revealing a mechanistic connection between macrophage polarization and uric acid-driven inflammation [8, 9]. The phenotype and immune function of macrophages are intricately tied to their metabolic programming. M1 polarization predominantly relies on glycolysis, while M2 polarization requires fatty acid oxidation (FAO) [10]. Targeting the metabolic rewiring process to transform macrophage phenotypic polarization has garnered mounting attention as a promising avenue for gouty arthritis therapy [3, 11]. Increasing evidence suggests that macrophage activation dynamics can be orchestrated by multiple factors, including altered intestinal microbiota composition and its metabolites [12]. Aberrant gut microbiota composition, metabolites, and signaling have been shown to be associated with both local and systemic metabolic reprogramming [13]. Earlier work from our group, and others established the pathogenic link between gut microbiota dysfunction and gout [14, 15]. Specifically, depletion of short-chain fatty acid-producing bacteria, particularly butyric acid-producing bacteria (BPB), has been documented as a distinctive feature in the pathogenesis of gout [16, 17]. Thus, precision modulation of gut microbiota structure and metabolic output to skew macrophage polarization represents a novel therapeutic paradigm for gout management.

Traditional Chinese medicine (TCM), with millennia of therapeutic application in China for multifactorial disorders, is increasingly recognized in global healthcare systems. Qu-zhuo-tong-bi decoction (QZTBD), a patented nine-herb formula (Table S1; Patent No. ZL201010209598.7; Production certification Z20230015000), is clinically validated for managing hyperuricemia and gouty arthritis. QZTBD exerts a substantial protective impact on both articular and systemic inflammation, demonstrating comparable therapeutic efficacy to benzbromarone [17, 18]. Our prior work revealed that QZTBD restructures gut microbiota, enhances intestinal barrier integrity, and upregulates GPR43 expression in diet-induced and MSU-challenged murine models of gouty arthritis [18]. However, the exact mechanisms by which QZTBD achieves its efficacy through gut microbiota modulation remain to be elucidated, as do its immunomodulatory effects on macrophages.

In the present study, we explore the mechanisms of QZTBD in alleviating gouty arthritis using an integrated approach combining network pharmacology with in vivo and in vitro experimental validation. We demonstrate the potential role of glycolysis inhibition in attenuating gout progression by reprogramming macrophage polarization in *Uox*-KO mice. We also unveil the intricate interplay between TCM, gut microbiome dynamics, and host immune homeostasis, with a specific focus on macrophage phenotypic modulation.

## Materials and methods

### Medicine preparation and component analysis of QZTBD

QZTBD comprises nine traditional Chinese herbs, as detailed in Table S1. The formula was procured from Zhejiang Chinese Medical University Medical Pieces, Ltd. (Hangzhou, China). The chemical composition of the QZTBD aqueous extract was analyzed using a UHPLC-MS/MS system (Thermo Fisher Scientific) via an untargeted metabolomics approach. Samples were extracted with methanol/water (4:1, v/v) containing 10 μg/mL internal standard, followed by sonication, centrifugation, and filtration. Chromatographic separation was achieved on a Waters ACQUITY UPLC BEH C18 column (1.7 μm, 2.1 × 100 mm) with a mobile phase composed of 0.1% formic acid in water and 0.1% formic acid in acetonitrile. MS data were acquired in both positive and negative ionization modes and processed using the XCMS software. Detailed parameters and data analysis workflows are provided in Supplementary Methods.

### Animals and treatment

Male *Uox*-KO mice (3–5 weeks old), characterized by stably elevated serum uric acid levels [15, 19], were used in this study. Mice were housed in a specific pathogen-free barrier facility at the Laboratory Animal Center of Zhejiang Chinese Medical University (12 h light/dark cycle, 20 ± 2 °C, 60 ± 5% humidity). All experimental protocols were approved by the Institutional Animal Care and Use Committee (IACUC) of Zhejiang Chinese Medical University (Approval No. IACUC-20210621-17; June 2021).

*Uox*-KO mice were randomly assigned into experimental groups, with wild-type (WT) mice as controls. The QZTBD group received QZTBD via oral gavage once per day for six weeks at low (QZTBD-L, 9.0 g/kg/d), medium (QZTBD-M, 18.0 g/kg/d), and high (QZTBD-H, 36.0 g/kg/d) doses. The benzbromarone (BBR) group was administered 6.5 mg/kg/d benzbromarone (50 mg/tablet, Batch: J20180056; Kunshan Longdeng Ruidi Pharmaceutical Co., Ltd.) for six weeks as a positive control. The butyric acid-producing bacteria (BPB) group was administered 10^9^ CFU/mL butyric acid-producing bacteria suspension daily, and the butyrate group was given 500 mg/kg sodium butyrate (Sigma-Aldrich, USA) for six weeks. For glycolysis inhibition, 2-Deoxy-D-glucose (2-DG) dissolved in sterile phosphate-buffered saline (PBS) (pH 7.4) was intraperitoneally injected at a dose of 250 mg/kg every other day for 6 weeks. Vehicle control groups received equivalent volumes of sterile water through corresponding routes. Twenty-four hours before euthanasia, *Uox*-KO mice received intraplanar injection of 1 mg MSU crystals in 40 μL of PBS, while WT mice received PBS alone. The levels of uric acid (UA), alanine aminotransferase (ALT), aspartate aminotransferase (AST), blood urea nitrogen (BUN), creatinine (CREA) were determined using a Hitachi 3100 analyzer (Tokyo, Japan). Paw swelling index and mechanical pain threshold were evaluated as previously described [17, 18].

### Cell culture and treatment

Primary bone marrow-derived macrophages (BMDMs) were isolated from the femurs and tibias of C57BL/6 J mice and cultured in RPMI-1640 medium (Thermo Fisher Scientific, USA) supplemented with 10% fetal bovine serum (FBS), 1% penicillin–streptomycin, and 25 ng/mL macrophage colony-stimulating factor (M-CSF). Cells were maintained for 7 days in a humidified 5% CO_2_ incubator at 37 °C and adherent cells were collected as BMDMs. For M1 polarization, M0 macrophages were treated with pharmacological interventions, including QZTBD-containing serum, butyrate (0.5 mM, 1.0 mM, or 1.5 mM) [20, 21], or 2-DG (1.5 mM), 24 h prior to harvest. 100 ng/mL lipopolysaccharide (LPS) and 50 ng/mL interferon-γ (IFN-γ) were added 6 h before harvest to induce polarization.

### Fecal microbiota transplantation

Fecal microbiota transplantation (FMT) was carried out following the methodology described in the previous study [18]. Briefly, fresh fecal pellets from QZTBD-treated donor mice were promptly collected each morning post defecation. 200 mg fecal pellets were suspended in 1 mL sterile PBS and centrifuged at 800 rpm for 5 min to remove debris. The collected supernatant was centrifuged at 12,000 rpm for 5 min to pellet microbial biomass. The resulting microbial pellet was then resuspended in PBS and administered via oral gavage to recipient *Uox*-KO mice (200 µL/mouse) within 10 min of preparation. This procedure was repeated daily for six weeks, with recipients designated as the QZ-FMT group.

### Analysis of QZTBD on gut microbiota activity

Fresh fecal microbiota samples from mice were collected and anaerobically cultured in MRS broth using an anaerobic chamber (80% N_2_, 10% H_2_, and 10% CO_2_) at 37 °C with 60% relative humidity. The microbiota was treated with different doses of QZTBD (0, 2.5, 5, and 10%) for 24 h. Microbial biomass was quantified spectrophotometrically at OD_600_, while butyrate kinase (*Buk*) and butyryl-CoA: acetyl-CoA transferase (*But*) gene expression levels were examined by RT-qPCR.

### Histopathology and immunofluorescence

Paraffin-embedded samples were prepared as previously described [17] and sectioned into 5-µm-thick sections. Tissue sections were deparaffinized in xylene, rehydrated through a graded ethanol series, stained with H&E, and imaged using bright-field microscope. For macrophage phenotyping, immunofluorescence (IF) staining was performed on claw tissue sections. Antibodies directed against F4/80, iNOS, and CD206 were utilized, and an anti-fluorescence quenching sealer containing DAPI was employed (Table S2). Fluorescence images were acquired using a confocal scanning microscope and fluorescence intensity quantification was analyzed using the ImageJ software.

### Pathway enrichment analysis of QZTBD

To ascertain the key signaling pathways associated with the active components of QZTBD, KEGG pathway enrichment analysis was carried out using the Metascape platform (http://metascape.org/). The results were subsequently subjected to a visual analysis using R 4.0.0. A significance level of *P* < 0.05 was established for the screening.

### 16S rRNA amplicon sequencing

Microbial genomic DNA was extracted from fecal samples utilizing the QIAamp DNA Microbiome Kit (Qiagen, Germany). The V3–V4 region of bacterial 16S rRNA was amplified and sequenced using the 2 × 250 bp paired-end reading on the Illumina NovaSeq PE250 platform. Raw sequencing data were demultiplexed, quality-filtered, and assembled. High-quality sequences were clustered into operational taxonomic units (OTUs) at a similarity threshold of 97% using UPARSE. All raw sequences were deposited in the Sequence Read Archive with individual sample accessions ranging from SAMN43357155 to SAMN43357181 and from SAMN31530587 to SAMN31530614.

### Untargeted serum metabolomics analysis

Serum samples (50 µL) were extracted with methanol/acetonitrile (1:1, v/v) containing internal standards, vortexed, and filtered. Quality control (QC) samples were prepared by pooling equal aliquots of all filtrates. Metabolomic profiling was performed using UHPLC (Vanquish, Thermo) coupled to an Orbitrap Exploris 120 MS. Chromatographic separation was conducted with two complementary columns. The first was a BEH Amide column (2.1 × 50 mm, 1.7 µm), utilizing a mobile phase composed of ammonium acetate/ammonium hydroxide (pH 9.75) and acetonitrile. The second was a Phenomenex Kinetex C18 column (2.1 × 100 mm, 2.6 µm), with a mobile phase consisting of 0.01% acetic acid aqueous solution and a mixture of isopropanol and acetonitrile (1:1, v/v). MS data were acquired in IDA mode (ESI: ± 3.8/3.4 kV, 60,000 resolution). Data were processed via XCMS (R-based), and metabolites were identified using BiotreeDB (V3.0).

### RT-qPCR

Total RNA was isolated from murine colon tissues and BMDMs using TRIzol reagent (Invitrogen, USA). cDNA was synthesized using the HiFiScript^®^ cDNA Synthesis Kit (Cwbio, China). Fecal microbial RNA was extracted from the mouse fecal pellets and then converted to cDNA with the use of SuperScript^™^ IV VILO^™^ Master Mix (Thermo Fisher Scientific, USA). In addition, genus-specific qPCR primers were used to quantify the relative abundance of the representative butyrate-producing genera in QZ-FMT recipient mice. The results were normalized to 16S rRNA gene levels and calculated using 2^−ΔΔCt^ method. Primer sequences are listed in Table S3.

### Western blot analysis

Total protein was extracted from murine colon tissues and BMDMs using RIPA lysis buffer (Cat# R0020, Solarbio). Protein was quantified by BCA assay (Beyotime, # p0012). The extract was subjected to SDS-PAGE and subsequently electroblotted onto PVDF membranes. The proteins were probed with the specific primary antibodies (Table S4) and HRP-conjugated secondary antibody (Abcam, # ab205718, 1:5000). Protein signals were visualized with ECL system, and the gray value was quantified as a ratio to β-actin using ImageJ software.

### Meta-analysis of GEO dataset

The GSE160170 microarray datasets of human gouty arthritis was retrieved from the public GEO database (http://www.ncbi.nlm.nih.gov/geo/) at the National Center for Biotechnology Information (NCBI). Differentially expressed genes between healthy controls and gouty arthritis patients were identified using GEO2R online tools. Absolute log2-fold change > 1 and adjusted *P*-value < 0.05 were set as the cutoff criteria.

### Seahorse XF glycolysis stress assay

To evaluate the glycolytic capacity of BMDMs, the glycolysis stress test assay was performed using the Seahorse XF Glycolytic Rate Assay Kit (Agilent). Prior to the assay, BMDMs were seeded in XFp microplates at 20,000 cells/well and pre-treated with QZTBD-containing serum for 24 h under standard culture conditions. Extracellular acidification rate (ECAR) and oxygen consumption rate (OCR) were quantified using the Seahorse XFp Analyzer. A detailed and comprehensive account of the ECAR and OCR process can be found in the supplementary materials.

### Flow cytometry

Single-cell suspensions from BMDMs, spleens, and intestines were prepared as previously described [15, 18]. Cells were incubated with Fc block (anti-mouse CD16/32) to reduce nonspecific binding, followed by staining with fluorochrome-conjugated antibodies against CD45, F4/80, CD86, and CD163 (Table S5). Stained cells were washed with PBS and analyzed on a CytoFLEX S flow cytometer (Beckman Coulter, USA). Data were analyzed with FlowJo software (10.4.0). M1 macrophages were gated as CD45^+^ F4/80^+^ CD86^+^ cells, while M2 macrophages were gated as CD45^+^ F4/80^+^ CD163^+^ cells.

### Butyrate detection

Gas chromatography-mass spectrometry (GC-MS) was used for butyrate detection, and GPC-GC/MS-2010 (Shimadzu, Japan) with a Rtx-Wax capillary column (Restek, USA) was employed for analysis. Detailed protocols are provided in the Supplementary Materials.

### Enzyme-linked immunosorbent assay

The levels of cytokines IL-1β, IL-6, IL-18, TNF-α, and IL-10 in serum, colon tissue homogenates, and the culture supernatant of BMDMs were quantified by ELISA kits (Lianke, China) in accordance with the manufacturer's instructions.

### Preparation of butyric acid-producing bacteria

Butyric acid-producing bacterium (BPB, *Clostridium butyricum*) was incubated in MRS broth at 37 °C under 60% relative humidity for 24 h using a GeneScience E500 anaerobic chamber. Bacterial pellets were harvested by centrifugation and subjected to 3 washes with sterile PBS. The final biomass was resuspended in PBS to achieve a concentration of 10^9^ CFU/mL.

### Preparation of the QZTBD-containing serum

SD rats were used for the preparation QZTBD-containing serum (Approval No.: IACUC-20240219-15). A concentrated QZTBD solution (18.9 g/kg/d) was administered orally at a dose of 10.0 mL/kg for seven consecutive days. The control group was treated with 10.0 mL/kg saline to prepare blank serum. Blood samples were collected from the abdominal aorta and incubated at 37 °C for 30 min. Serum was separated by centrifugation at 3000 rpm and 4 °C for 15 min, heat-inactivated at 56 °C for 30 min, then filtered and sterilized through 0.22 μm microporous membranes.

### Molecular docking analysis

For molecular docking analysis, the 2D structures of the components were retrieved from the PubChem database (https://pubchem.ncbi.nlm.nih.gov/). The 3D crystal structures of *Buk* (PDB code: AF-Q05619-F1) and *But* (PDB code: AF-A0A073KPY3-F1) were obtained from the Protein Data Bank. PyMOL 2.1 software was employed to remove ligands, perform dehydration, and add hydrogens to the target protein. The target protein and main components were converted to PDBQT format using AutoDock Tools 4.2.6. The center coordinates and dimensions of the docking box were defined based on the protein active site and the ligand binding region. Molecular docking was conducted utilizing AutoDock Vina 1.1.2 software, and the results were expressed as docking scores.

### Statistical analysis

The data analysis was conducted using GraphPad Prism 9.0.2 software (GraphPad Software, USA), with data expressed as mean ± standard error of the mean (SEM). For comparisons involving multiple groups, one-way ANOVA followed by Tukey test was used to determine statistical significance. Spearman's correlation analysis was employed to investigate potential relationships between parameters. Each experiment was conducted with at least three replicates. No data points were excluded except in cases of technical failure. Statistical significance was defined as* P* < 0.05, with the following notation: *P < 0.05, **P < 0.01, ***P < 0.001, and ns (not significant).

## Results

### QZTBD alleviates gouty arthritis in *Uox*-KO mice

Successful establishment of the gouty arthritis model in *Uox*-KO mice was confirmed through comprehensive phenotypic and biochemical analyses. *Uox*-KO mice displayed significantly elevated serum UA levels compared to WT controls (Fig. S1A), along with characteristic joint swelling, histological evidence of inflammatory infiltration, and reduced pain thresholds (Fig. S1B-D), confirming the recapitulation of hyperuricemia and inflammatory joint pathology typical of gouty arthritis.

**Fig.1:**
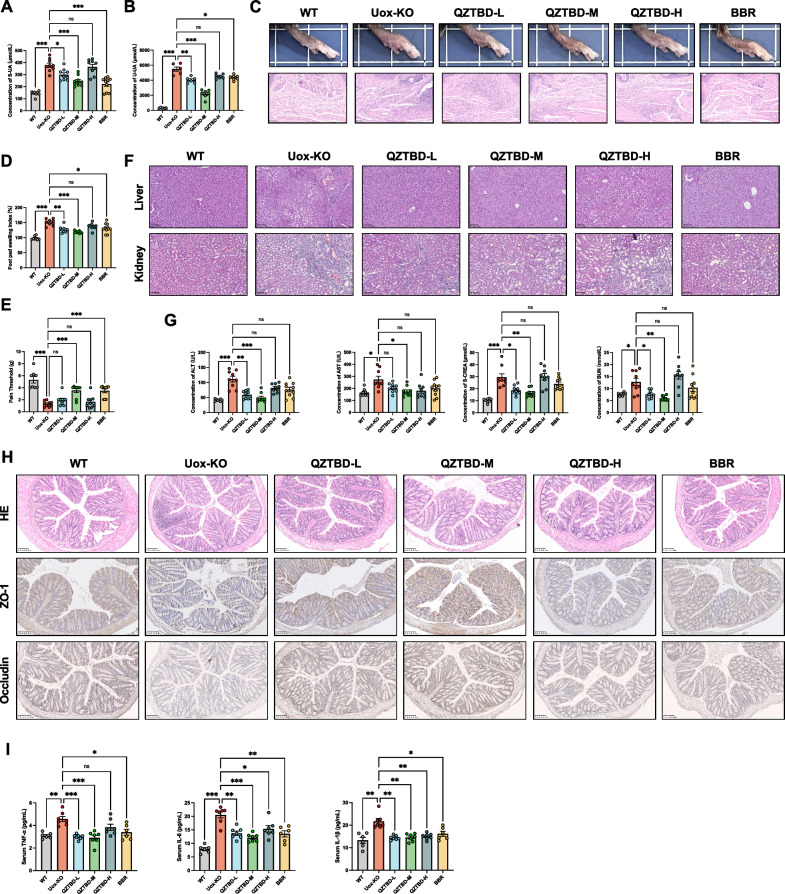
Therapeutic effects of QZTBD on disease symptoms of *Uox*-KO mice. **A**, **B** Serum and urinary UA levels after QZTBD and BBR treatment. **C** Representative images and H&E-stained sections of footpads 24 h post-MSU injection (Scale bar: 100 µm). **D**, **E** Footpad swelling index and mechanical pain threshold after treatment. **F** H&E-stained liver (top) and kidney (bottom) histological sections (Scale bar: 100 µm). **G** QZTBD (18.0 g/kg/d) attenuated hepatic and renal pathological damage. **H** Colon sections stained with H&E (top) and IHC staining for ZO-1 (middle) and occludin (bottom) (Scale bar: 100 µm). **I** Serum IL-1β, IL-6, and TNF-α concentrations measured by ELISA (n = 6). n = 7–9 mice per group. Values are expressed as mean ± SEM. ns, not significant; *P < 0.05, **P < 0.01, ***P < 0.001

Therapeutic evaluation over a 6-week treatment period revealed dose-dependent effects of QZTBD in *Uox*-KO mice. Medium-dose QZTBD (18.0 g/kg/d) exhibited the most significant uric acid-lowering effects (Fig. 1A, B) and anti-inflammatory activity (Fig. 1C–E), comparable to those of BBR. Furthermore, 18.0 g/kg/d QZTBD showed a satisfactory safety profile and effectively restored impaired liver, spleen, and kidney indices (Fig. S2A-D). Histopathological analysis revealed that 18.0 g/kg/d of QZTBD efficiently attenuated hepatic inflammatory infiltration, renal tubular atrophy, and glomerular fibrosis in *Uox*-KO mice (Fig. 1F). Blood biochemical analysis suggested marked reductions in ALT, AST, S-CREA, and BUN levels in *Uox*-KO mice treated with 18.0 g/kg/d QZTBD, demonstrating hepatoprotective and renoprotective effects not observed with BBR treatment (Fig. 1G). We also assessed the intestinal inflammation and integrity using HE and IHC analysis, respectively. 18.0 g/kg/d QZTBD treatment effectively improved the colonic morphology of *Uox*-KO mice, upregulated tight junction protein expression, and alleviated intestinal inflammation (Fig. 1H). Additionally, ELISA results confirmed that 18.0 g/kg/d QZTBD suppressed systemic inflammation in *Uox*-KO mice by decreasing serum levels of TNF-α, IL-6, and IL-1β (Fig. 1I). These findings, combined with our previous studies [17, 18], validate the therapeutic potential and safety profile of 18.0 g/kg/d QZTBD for gouty arthritis, establishing this dose as optimal for the subsequent investigations.

### Components analysis and target prediction of QZTBD

**Fig.2:**
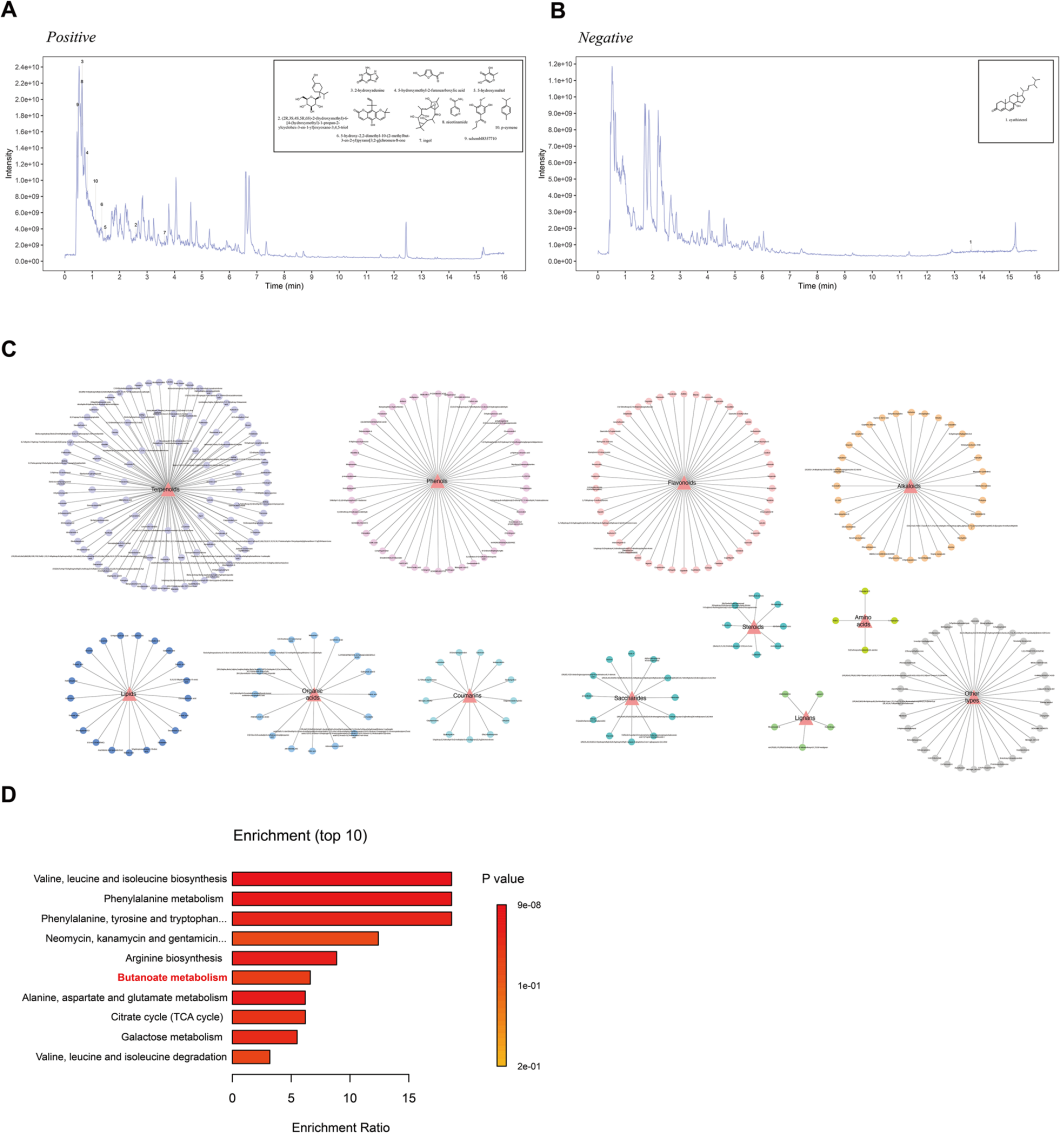
Chemical profiling of QZTBD aqueous extracts by UHPLC-MS/MS. **A** Total ion chromatography in positive ion mode. **B** Total ion chromatography in negative ion mode. **C** Classification of the 344 identified or tentatively characterized compounds. **D** KEGG pathway enrichment analysis of QZTBD bioactive components

Chemical profiling of QZTBD was performed using UHPLC-MS/MS. Representative base peak chromatograms in positive and negative ion modes were shown in Fig. 2A, B, with the top 10 compounds exhibiting the highest peak intensities and composite scores annotated (Table S6). A total of 344 chemical constituents were identified or tentatively characterized, comprising 107 terpenoids, 46 phenols, 46 flavonoids, 32 alkaloids, 21 lipids, 16 organic acids, 13 coumarins, 12 saccharides, 8 steroids, 5 lignans, 4 amino acids, and 34 other compounds (Fig. 2C). Detailed compound annotations were provided in Table S7. Metabolite enrichment analysis revealed significant pathway enrichment in amino acid metabolism, butanoate metabolism, and the citrate cycle (TCA cycle) (Fig. 2D).

### QZTBD regulates macrophage polarization

**Fig.3:**
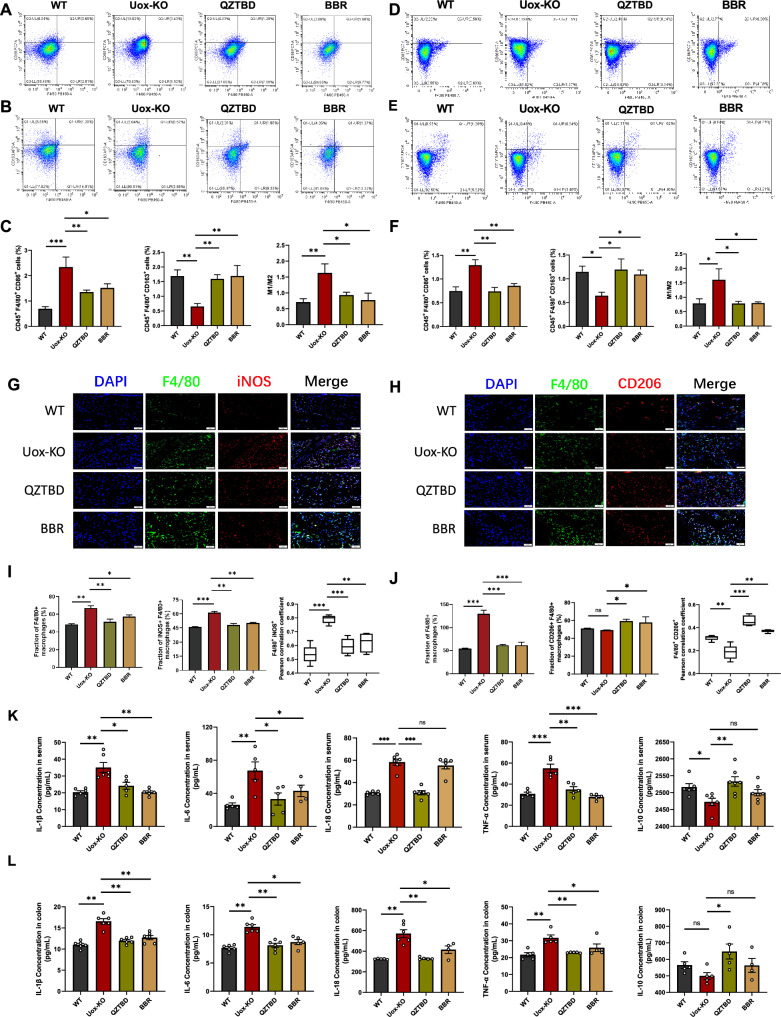
QZTBD restores M1/M2 macrophage balance in *Uox*-KO mice. **A**, **B** Representative flow cytometry plots of intestinal lamina propria M1 macrophages (CD86^+^ F4/80^+^ CD45^+^) and M2 macrophages (CD163^+^ F4/80^+^ CD45^+^). **C** Percentage of intestinal M1 and M2 macrophages and M1/M2 ratio (n = 7). **D**, **E** Representative flow cytometry plots of splenic M1 and M2 macrophages. **F** Percentage of splenic M1 and M2 macrophages and M1/M2 ratio (n = 7). **G**–**J** Immunofluorescence of M1 and M2 macrophages in footpad tissue sections (Scale bar: 50 μm) (n = 4). **K**, **L** Cytokine levels (IL-1β, IL-6, TNF-α, IL-18, IL-10) in serum and intestinal tissue (n = 5). Values are expressed as mean ± SEM. ns, not significant; *P < 0.05, **P < 0.01, ***P < 0.001

Given that macrophage-mediated inflammatory responses represent the most basic and fundamental process in the pathogenesis of gouty arthritis pain [22], we investigated the effects of QZTBD on macrophage polarization. Flow cytometry results demonstrated that QZTBD exerted a profound inhibitory effect on the polarization of M1 macrophages in the intestine and spleen, and promoting M2 polarization, thereby restoring the M1/M2 balance (Fig. 3A–F). Immunofluorescence staining revealed increased mean fluorescence intensity of M1 macrophages (F4/80^+^ iNOS) in the footpad of *Uox*-KO mice, concomitant with decreased M2 macrophages (F4/80^+^ CD206). QZTBD treatment induced a phenotypic shift from M1 to M2 macrophages (Fig. 3G–J). ELISA analysis showed that QZTBD downregulated pro-inflammatory cytokines, including IL-1β, IL-6, IL-18, and TNF-α, and upregulated the anti-inflammatory cytokine IL-10 in both serum and intestine (Fig. 3K, L). Our findings suggest that QZTBD modulates macrophages plasticity by inhibiting M1 polarization, rebalancing macrophage subsets, and attenuating inflammatory cascade response.

### QZTBD modulates butanoate metabolism

**Fig.4:**
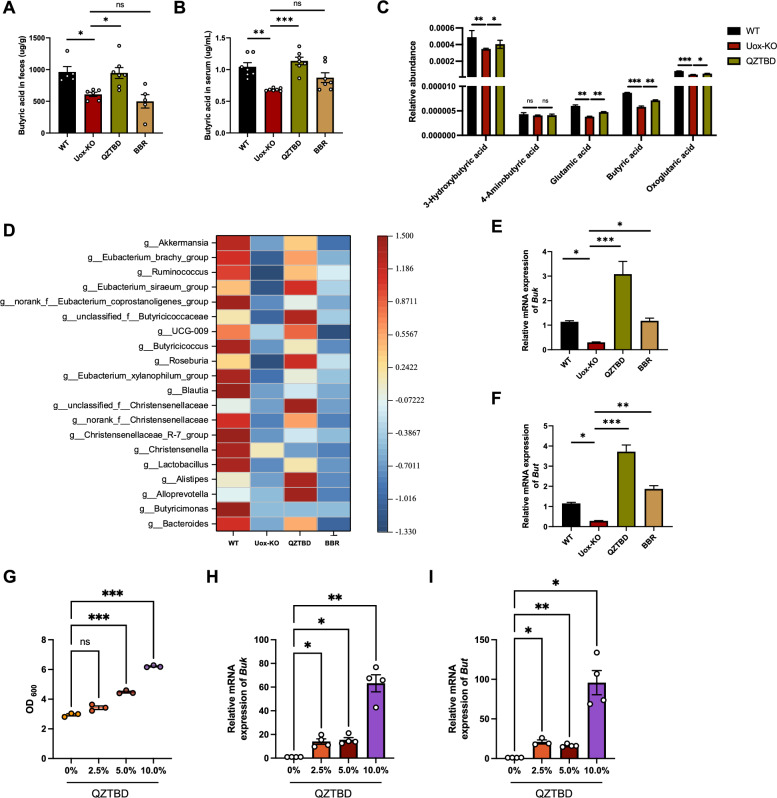
QZTBD regulates butyrate metabolism in *Uox*-KO mice. **A**, **B** Fecal and serum butyric acid levels after QZTBD and BBR treatment (n = 5–7). **C** Relative abundance of metabolites in the butyrate metabolism pathway post-QZTBD treatment (n = 6). **D** Relative abundance of butyric acid-producing bacteria (n = 7). **E**, **F** Relative expression of *Buk* and *But* in intestinal contents after treatment (n = 7). **G** Bacterial growth density measured by OD_600_ (n = 3). **H**, **I** Relative expression of *Buk* and *But* in bacteria cultured with QZTBD (n = 3–4). Values are expressed as mean ± SEM. ns, not significant; *P < 0.05, **P < 0.01, ***P < 0.001

To investigate the effect of QZTBD on butanoate metabolism, we first assessed the serum and fecal butyric acid levels in *Uox*-KO mice by GC-MS. Untreated *Uox*-KO mice exhibited markedly reduced butyric acid levels in both serum and feces, while QZTBD administration restored these levels to WT-equivalent concentrations, which was not observed with BBR treatment (Fig. 4A, B). Furthermore, untargeted serum metabolomics focusing on the butanoate metabolism pathway (KEGG pathway ko00650) identified five key intermediates, four of which—3-hydroxybutyric acid, glutamic acid, butyric acid, and oxoglutaric acid—were significantly upregulated after QZTBD treatment (Fig. 4C).

Since butanoate metabolism is tightly regulated by gut microbiota, 16S rRNA sequencing was conducted and the abundance of butyric acid-producing bacteria (BPB) was analyzed. A significant depletion of BPB was observed in *Uox*-KO mice, which was reversed by QZTBD intervention (Fig. 4D). We further investigated the expression of butyrate kinase (*Buk*) and butyryl-CoA: acetyl-CoA transferase (*But*), enzymes that play a pivotal role in butyric acid metabolism, by RT-qPCR. The results showed that the expression of *Buk* and *But* was considerably decreased in *Uox*-KO mice. Administration of QZTBD effectively restored the expression of these enzymes (Fig. 4E, F). In addition, we isolated the fresh fecal bacteria from mouse feces and cultured them under anaerobic conditions with different concentrations of QZTBD for 24 h. The results showed that QZTBD dose-dependently enhanced bacterial growth and upregulated *Buk* and *But* expression (Fig. 4G, I). Taken together, these results established QZTBD as a potent modulator of bacterial butanoate metabolism.

### Supplementation of BPB and butyrate alleviates gouty arthritis

**Fig.5:**
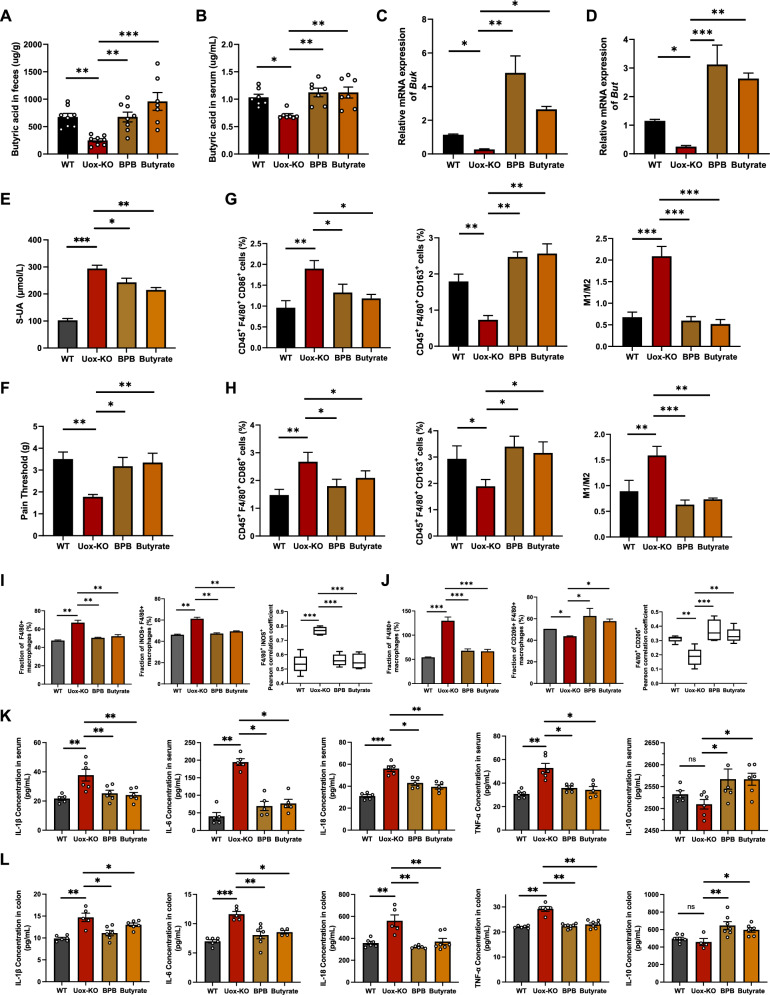
Effects of BPB and butyrate supplementation on macrophage polarization. **A**, **B** Fecal and serum butyric acid after BPB and butyrate treatment (n = 7–8). **C**, **D** Relative mRNA expression of *Buk* and *But* expression after BPB and butyrate treatment (n = 7). **E** Serum UA levels after BPB and butyrate treatment (n = 6–7). **F** Mechanical pain threshold after BPB and butyrate treatment (n = 6–7). **G** Percentage of intestinal M1 and M2 macrophages and M1/M2 ratio after BPB and butyrate treatment (n = 7). **H** Percentage of splenic M1 and M2 macrophages and M1/M2 ratio after BPB and butyrate treatment (n = 7). **I**, **J** Immunofluorescence of M1 and M2 macrophages in footpad tissue after BPB and butyrate treatment (n = 4). **K**, **L** Cytokine levels in serum and intestinal tissue after BPB and butyrate treatment (n = 5–6). Values are expressed as mean ± SEM. *P < 0.05, **P < 0.01, ***P < 0.001

To investigate the critical role of BPB in QZTBD-mediated alleviation of gouty arthritis, *Uox*-KO mice received daily oral gavage of BPB or butyrate for six weeks. Both interventions rescued impaired butyrate metabolism in *Uox*-KO mice, as evidenced by elevated fecal and serum butyric acid levels (Fig. 5A, B) and restored expression of *Buk* and *But* (Fig. 5C, D). This restoration resulted in a notable reduction in uric acid levels (Fig. 5E) and a discernible alleviation of inflammatory cell infiltration in footpads (Fig. 5F, Fig. S3A-B). The administration of BPB and butyrate also reprogrammed macrophage polarization in intestinal, splenic, and footpad tissues, increasing M2 macrophage proportions and reducing M1/M2 ratios (Fig. 5G–J, Fig. S3C-F). Furthermore, the expression of pro-inflammatory cytokines, including IL-1β, IL-6, IL-18, and TNF-α, was dramatically decreased following BPB and butyrate treatment, while the level of anti-inflammatory cytokine IL-10 was markedly increased (Fig. 5K, L). These findings suggest that BPB and butyrate supplementation recapitulates the therapeutic effects of QZTBD by restoring butyrate metabolism and polarizing macrophages towards an anti-inflammatory phenotype.

### QZTBD skews macrophage polarization by modulating intestinal flora

**Fig.6:**
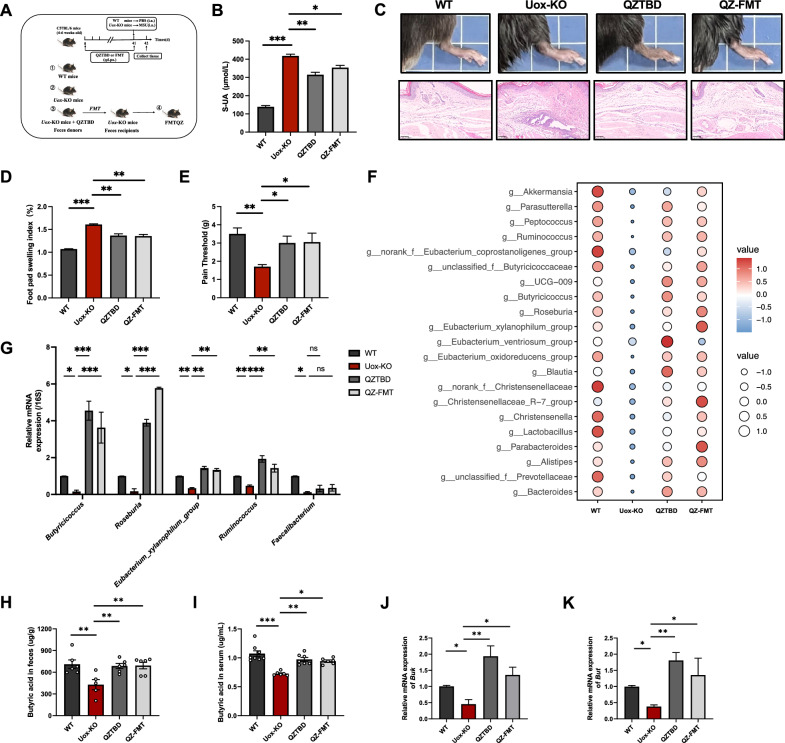
Fecal microbiota transplantation regulates butyrate metabolism in *Uox*-KO mice. **A** Schematic of FMT experimental design. **B** Serum UA level after QZTBD and QZ-FMT treatment. **C**, **D** Footpad swelling index after QZTBD and QZ-FMT treatment. **E** Mechanical allodynia thresholds after QZTBD and QZ-FMT treatment. **F** Relative abundance of the butyric acid-producing bacteria after QZTBD and QZ-FMT treatment. **G** qPCR quantification of representative butyrate-producing bacteria after QZTBD and QZ-FMT treatment. **H**, **I** Fecal and serum butyric acid levels after QZTBD and QZ-FMT treatment. **J**, **K** Relative mRNA expression of *Buk* and *But* after QZTBD and QZ-FMT treatment. n = 6–8 mice per group. Values are expressed as mean ± SEM. *P < 0.05, **P < 0.01, ***P < 0.001

To further ascertain the importance of BPB in QZTBD treatment, fecal microbiota transplantation was performed by transferring gut microbiota from the QZTBD-treated mice to *Uox*-KO mice (Fig. 6A). After six weeks of FMT, recipient mice (QZ-FMT) exhibited therapeutic outcomes mirroring direct QZTBD treatment, including a significant reduction in UA concentration (Fig. 6B), a marked decline in edema, and a robust anti-allodynic effect (Fig. 6C–E). 16S rRNA gene sequencing confirmed BPB enrichment in QZ-FMT mice (Fig. 6F), which was further validated by genus-specific qPCR targeting five representative butyrogenic genera, including *Roseburia*, *Butyricicoccus*, *Eubacterium_xylanophilum_*group, *Faecalibacterium,* and *Ruminococcus*. Four of these genera, excluding *Faecalibacterium,* exhibited significant expansion in both QZTBD-treated and QZ-FMT mice compared to untreated *Uox*-KO controls (Fig. 6G). This microbial remodeling was further evidenced by the elevated fecal and serum butyric acid levels (Fig. 6H, I) and the upregulated *Buk* and *But* expression in QZ-FMT mice (Fig. 6J, K).

**Fig.7:**
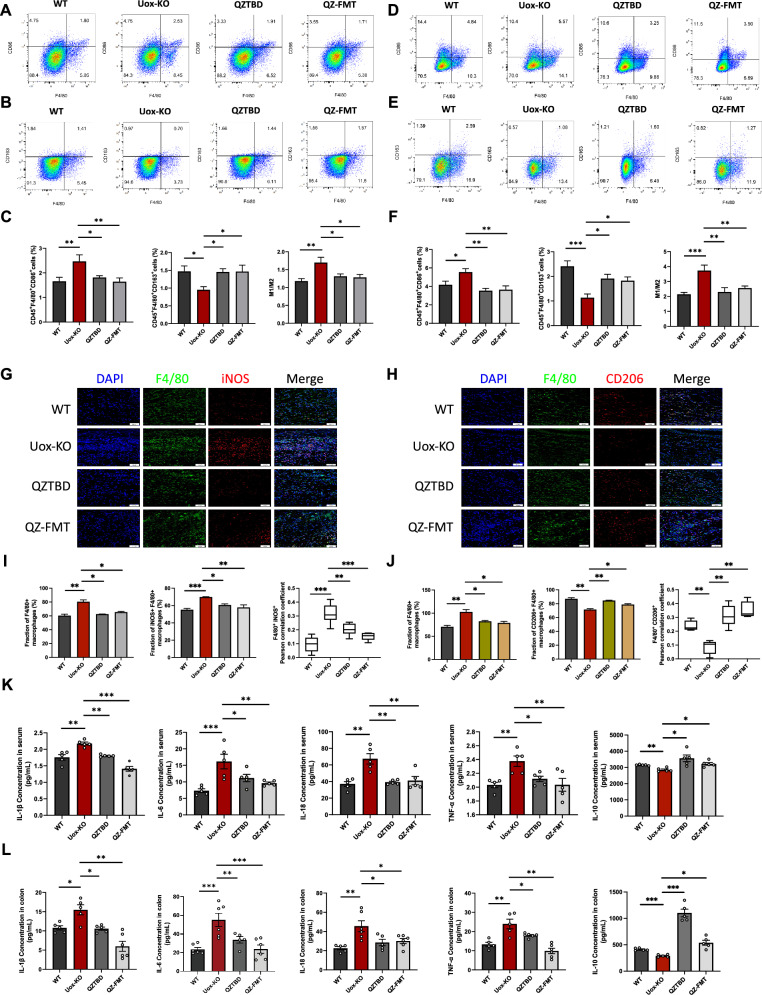
Fecal microbiota transplantation reprograms macrophage polarization in *Uox*-KO mice. **A**, **B** Representative flow cytometry plots of M1 macrophages and M2 macrophages in intestinal lamina propria. **C** Percentage of intestinal M1 and M2 macrophages and M1/M2 ratio (n = 7). **D**, **E** Representative flow cytometry plots of M1 and M2 macrophages in spleen. **F** Percentage of splenic M1 and M2 macrophage and M1/M2 ratio (n = 7). **G**–**J** Immunofluorescence of M1 and M2 macrophages in footpad (Scale bar: 50 μm) (n = 4). **K**, **L** Cytokine levels in serum and intestinal tissue (n = 5–6). Values are expressed as mean ± SEM. ns, not significant; *P < 0.05, **P < 0.01, ***P < 0.001

Macrophage profiling in QZ-FMT mice recapitulated donor phenotypes. Transplantation of QZTBD-treated microbiota effectively suppressed pro-inflammatory M1 polarization and enhanced anti-inflammatory M2 polarization in the intestine, spleen, and footpad (Fig. 7A–J). ELISA analysis revealed comparable patterns in the QZ-FMT mice and the QZTBD-treated mice (Fig. 7K, L). Altogether, our findings suggest that QZTBD exerts regulatory effects on macrophage polarization through its impact on the composition and metabolic activity of the intestinal flora.

### QZTBD and butyrate orchestrate metabolic profiling in macrophages

**Fig.8:**
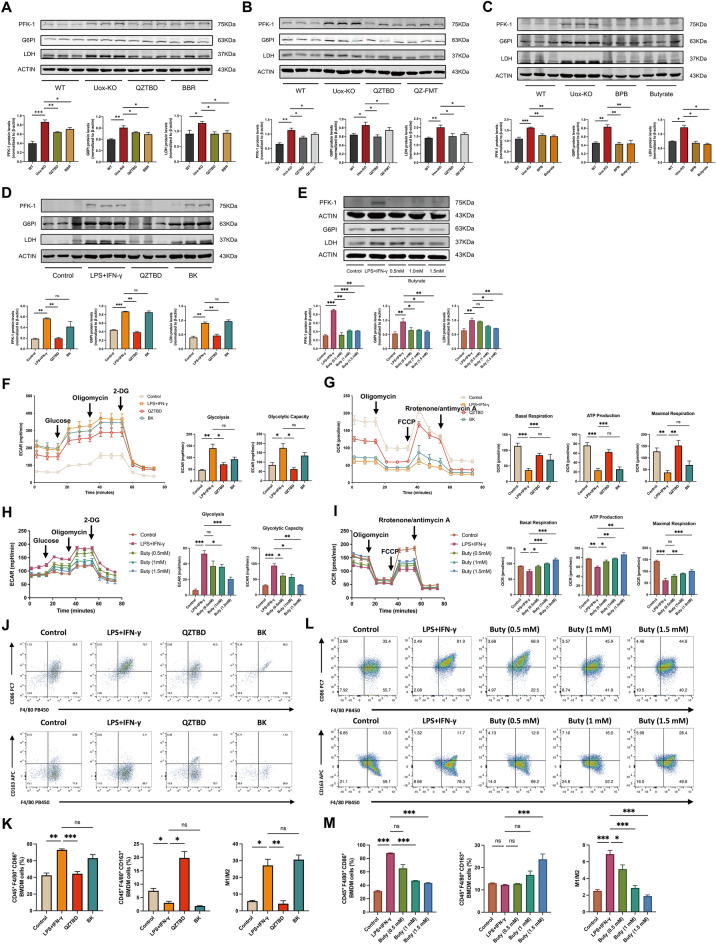
QZTBD and butyrate regulate macrophage metabolic reprogramming. **A** PFK-1, G6PI, and LDH protein expression in colon tissue after QZTBD and BBR treatment. **B** PFK-1, G6PI, and LDH protein expression in colon tissue post-QZTBD and QZ-FMT treatment. **C** PFK-1, G6PI, and LDH protein expression in the colon tissue following BPB and butyrate intervention. **D**–**E** PFK-1, G6PI, and LDH protein expression in BMDMs. **F**–**I** ECAR and OCR in BMDMs treated with QZTBD-containing serum (10%) and butyrate (0.5 mM, 1.0 mM, 1.5 mM) treatment. **J**–**M** Phenotypic shift from M1 to M2 macrophage after treatment with QZTBD-containing serum and butyrate in *vitro*. n = 3 mice per group. Values are expressed as mean ± SEM. ns, not significant; *P < 0.05, **P < 0.01, ***P < 0.001

It is well established that the activation of macrophages, similar to the Warburg effect in tumors, results in a metabolic shift towards glycolysis and away from oxidative phosphorylation (OXPHOS) [23]. Western blot analysis revealed the elevated expression of glycolytic enzymes, including PFK-1, G6PI, and LDH, in *Uox*-KO mice. However, QZTBD treatment (Fig. 8A) and fecal microbiota transplantation from QZTBD-treated mice (Fig. 8B) effectively reversed this phenomenon. Furthermore, BPB and butyrate treatment similarly inhibited the expression of these glycolytic enzymes (Fig. 8C). The same results were observed in vitro in BMDMs treated with QZTBD-containing serum (Fig. 8D) or butyrate (Fig. 8E). Taken together, these results suggest that BPB and butyrate, which can be modulated by QZTBD, may serve as efficient regulators of macrophage metabolic processes.

To better ascertain the effects of QZTBD and butyrate on macrophage metabolic phenotypes, we subsequently measured the extracellular acidification rate (ECAR) and oxygen consumption rate (OCR) of BMDMs following treatment with QZTBD-containing serum and butyrate. M1 polarization exhibited enhanced glycolytic capacity, accompanied by a significant reduction in basal respiration, adenosine triphosphate (ATP) production, and maximal respiration. QZTBD-containing serum reversed this metabolic imbalance by suppressing glycolysis and restoring OXPHOS (Fig. 8F, G). Butyrate dose-dependently reduced ECAR and elevated OCR (Fig. 8H, I), indicating metabolic reprogramming toward oxidative metabolism. Flow cytometry confirmed that QZTBD and butyrate treatment markedly impeded the differentiation of macrophages into pro-inflammatory M1 macrophages and facilitated the polarization of macrophages into anti-inflammatory M2 macrophages (Fig. 8J–M). ELISA analysis showed that IL-1β, IL-6, IL-18, and TNF-α were decreased after QZTBD and butyrate treatment, while the level of IL-10 was increased (Fig. S4A-B).

### Inhibition of glycolysis alleviates gouty arthritis

**Fig. 9:**
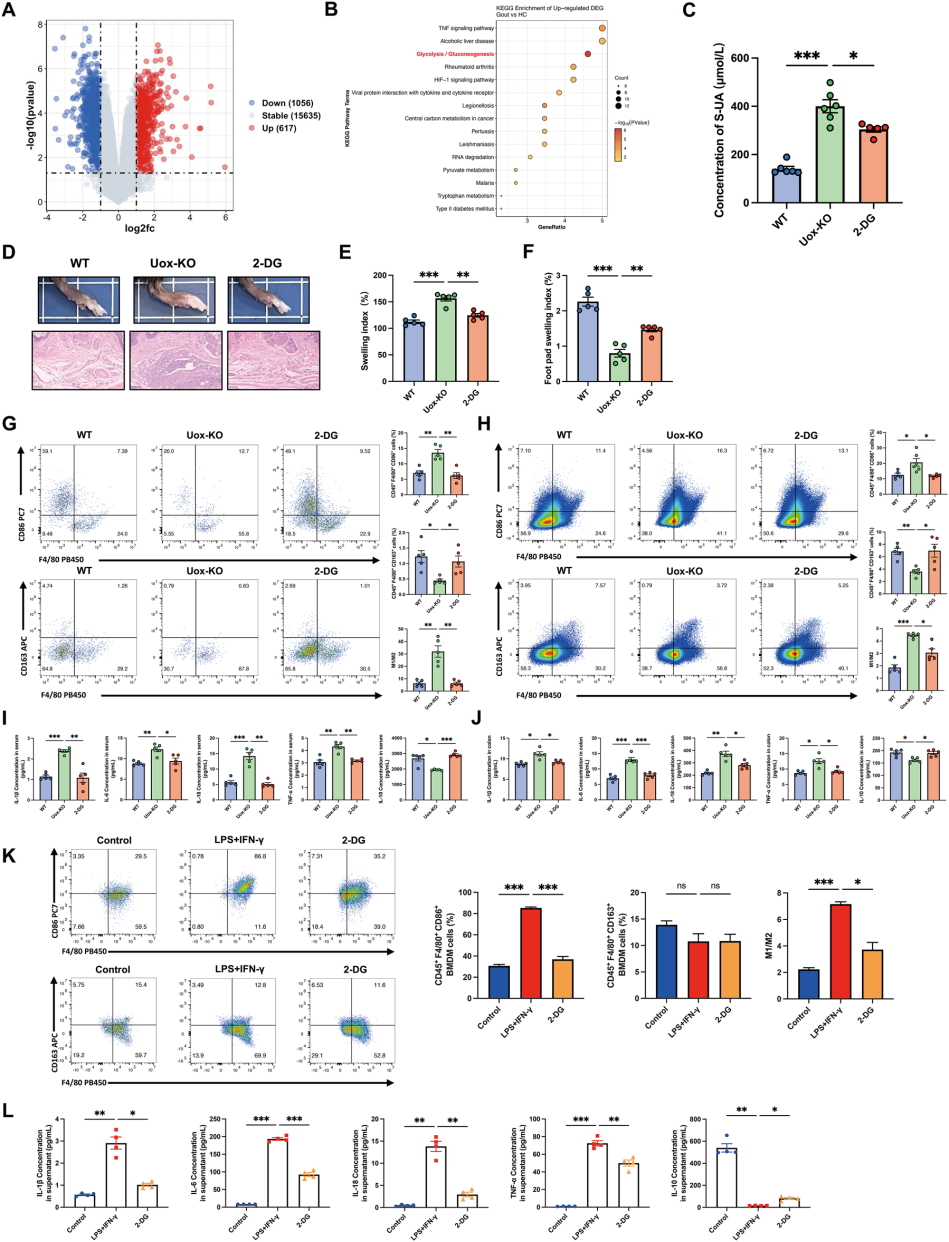
Glycolytic inhibition ameliorates macrophage polarization imbalance and gouty arthritis. **A** Volcano plot of differentially expressed genes (DEGs) in gout (n = 3). **B** KEGG pathway enrichment of upregulated DEGs in Gout. **C** Serum UA levels after 2-DG treatment (n = 5–6). **D**–**F** 2-DG attenuates joint inflammation in *Uox*-KO mice (Scale bar: 100 µm) (n = 5). **G**, **H** Representative flow cytometry plots, the percentage and the ratio of M1 and M2 macrophages in intestine and spleen after 2-DG treatment (n = 5). **I**, **J** Serum and intestinal cytokine levels after 2-DG treatment (n = 5). **K** In vitro M1/M2 polarization in BMDMs after 2-DG treatment (n = 4). **L** The levels of cytokines in BMDMs supernatant after 2-DG treatment (n = 4). Values are expressed as mean ± SEM. ns, not significant; *P < 0.05, **P < 0.01, ***P < 0.001

Given the established role of QZTBD in alleviating gouty arthritis through glycolysis-regulated macrophage polarization, we explored whether targeting glycolytic pathways could mitigate gout-associated inflammation. We first reanalyzed the microarray data downloaded from the GSE160170 dataset in the Gene Expression Omnibus (GEO) database. In comparison with healthy controls, 1,056 genes were downregulated and 617 genes were upregulated in gout patients (Fig. 9A). Subsequent KEGG pathway enrichment analysis identified glycolysis as a top upregulated pathway in gout patients (Fig. 9B). We further investigated the impact of glycolysis inhibition on macrophage polarization and gouty arthritis in *Uox*-KO mice using 2-deoxy-D-glucose (2-DG), a competitive inhibitor of glycolysis. The results showed that 2-DG administration reduced the level of UA (Fig. 9C), ameliorated arthritis-associated inflammation (Fig. 9D–F), restored M1/M2 macrophage balance (Fig. 9G, H), and suppressed the expression of pro-inflammatory cytokines (Fig. 9I–J). In addition, the influence of 2-DG on the metabolic profile of macrophages was examined in vitro. As anticipated, 2-DG supplementation elicited a significant reduction in glycolysis and an increase in OXPHOS in M1 macrophages (Fig. S5A). Consistently, the generation of M1 macrophages was successfully inhibited (Fig. 9K), and the secretion of cytokines was rebalanced by 2-DG treatment (Fig. 9L). Our results suggest that the inhibition of glycolysis is a crucial mechanism in the orchestration of macrophage polarization and the attenuation of inflammatory processes. Targeting glycolysis represents a promising strategy for the management of gouty arthritis.

## Discussion

In recent years, the rapid advancement of medical technology and the success of various animal models of gouty arthritis have facilitated the discovery of new drugs for treating gouty arthritis. However, these therapeutic strategies have been shown to target either the reduction of UA generation, the increase in its removal, the inhibition of its reabsorption, or the suppression of inflammatory factor expression. Few drugs have been found to reduce both UA levels and suppress inflammation. The development of medications capable of addressing both acute gouty arthritis inflammation and lowering uric acid levels is of paramount importance and is recommended as a therapeutic approach for gout.

Contrary to the widely accepted “one disease-one target-one drug” dogma in pharmaceutical development, TCM exhibits unique advantages and enhanced efficacy in treating complex diseases [24–28] through its intrinsic “multi-component, multi-target, and multi-pathway” mechanisms. QZTBD, a multi-herbal preparation with demonstrated efficacy and safety, has been developed as a novel targeted therapy for gouty arthritis [18]. The present study builds upon prior findings by providing additional evidence supporting QZTBD’s dual action in uric acid reduction and inflammation suppression, coupled with a favorable safety profile. However, unresolved ambiguities regarding its chemical composition and mechanistic underpinnings hinder clinical translation.

In the current study, UHPLC-MS/MS analysis identified 344 chemical components in QZTBD. Pathway enrichment analysis revealed that amino acid metabolism, butanoate metabolism, and citrate cycle were key pathways mediating the therapeutic effects of QZTBD. This aligns with our previous findings that QZTBD exerts its pharmacological effects against gout by modulating host amino acid metabolism and regulating CD4^+^ T cell differentiation [18]. The present work further explores the therapeutic mechanism of this empirical herbal formula, showing its ability to reprogram macrophage polarization via gut microbiota-dependent butanoate metabolism, thereby expanding the understanding of TCM in the regulation of host immunity and maintenance of intestinal homeostasis.

Growing evidence implicates gut microbiota dysbiosis in the pathogenesis of arthritic diseases, including gouty arthritis. Altered gut microbial composition and metabolic activity have been linked to impaired uric acid metabolism, heightened pro-inflammatory production, and intestinal barrier dysfunction [29]. In this study, *Uox*-KO mice exhibited reduced abundance of butyrate-producing bacteria, downregulated expression of *Buk* and *But,* and diminished fecal and serum butyrate levels. The alleviation of gout symptoms with BPB administration in our study underscores targeting BPB deficiency as a promising avenue for the treatment of gouty arthritis. These findings strongly support the crucial role of BPB in gout progression. In fact, clinical relevance is evidenced by reduced BPB abundance in gout patients [16]. Individuals with asymptomatic hyperuricemia have been found to possess a gut microbiota with a higher abundance of BPB than those with gout. This microbial profile has been linked to the production of anti-inflammatory mediators that may contribute to the prevention of gout flares [30]. Thus, modulating BPB through exogenous supplementation or endogenous enrichment represents a viable strategy for gouty arthritis management.

Numerous studies have shown that TCM can regulate the gut microbiome in a targeted and systematic manner to restore microbial homeostasis, repair intestinal barrier integrity, and ultimately prevent the development of the disease [31, 32]. The successful FMT results in this study corroborate the effect of QZTBD on the regulation of intestinal flora. Our prior work revealed that QZTBD alleviates gouty arthritis in high-fat diet (HFD)-induced mice by regulating gut microbial structure and restoring depleted butyrate level [17], though the precise molecular mechanism remained unclear. The present investigation demonstrated that treatment with QZTBD, but not BBR, attenuated dysbiosis of butyrate-producing bacteria in *Uox*-KO mice, including *Akkermansia*, *Eubacterium*, *Ruminococcus*, *Butyricicoccus*, *Roseburia*, *Lactobacillus*, *Alistipes*, *Alloprevotella,* and *Bacteroides*, highlighting the prebiotic potential of Chinese herbal medicines in the management of gouty arthritis.

It has been well documented that butyrate, an energetic metabolite produced by the intestinal bacteria, plays multifaceted roles in gastrointestinal physiology [33, 34]. In this study, butyrate emerged as the key mediator of QZTBD’s therapeutic effects, with exogenous administration effectively mitigating gout symptoms in *Uox*-KO mice. Several active ingredients in QZTBD possess the capacity to regulate bacterial butyrate metabolism. For example, nicotinamide (NAM), one of the most abundant constituents of QZTBD, serves as a precursor of nicotinamide riboside (NR). NR has been demonstrated to enhance the abundance of Firmicutes, a bacterial phylum known to produce butyrate, and to increase fecal butyrate levels [35, 36]. These findings underscore the efficacy of QZTBD in regulating gut microbiota and butanoate metabolism. In vitro experiments make it clear that QZTBD could directly promote the expression of butanoate metabolizing enzymes *Buk* and *Bu*t in a dose-dependent way, providing compelling evidence for the role of QZTBD as a prebiotic to foster the metabolic activities of beneficial bacteria and the regulatory effect on butanoate metabolism. What’s more, molecular docking analysis demonstrated strong binding affinity between QZTBD bioactive compounds and bacterial *Buk* and *Bu*t enzymes (Fig. S6A-D).

The crucial function of macrophages in orchestrating both the innate and adaptive immune responses emphasizes their substantial impact on the pathogenesis of gouty arthritis [31, 37]. Our discoveries unveil a distinctive alteration in macrophage polarization towards the pro-inflammatory M1 phenotype in *Uox*-KO mice, as indicated by an elevated M1/M2 ratio and enhanced glycolytic capacity. Blocking glycolysis with 2-DG resulted in impaired M1 macrophage polarization in vitro and eased the progression of gouty inflammation in vivo*.* Butyrate has been documented to mitigate the onset of acute gouty arthritis by inhibiting histone deacetylase and curbing the production of IL-1β, IL-6, and IL-8 in response to MSU [38]. However, the present study proposes that butyrate may be able to influence macrophage polarization by redirecting their metabolic profile from glycolysis towards OXPHOS. The suppressed expression of PFK-1, G6PI, and LDH and the inhibited extracellular acidification rates after QZTBD and butyrate treatment suggest the potential of QZTBD to modulate macrophage glycolysis by regulating butyrate production. Modulation of macrophage metabolism by gut microbiota-derived butyrate is a prospective therapeutic approach of QZTBD, and the “multi-component, multi-target” strategy is the core connotation of TCM.

**Fig. 10:**
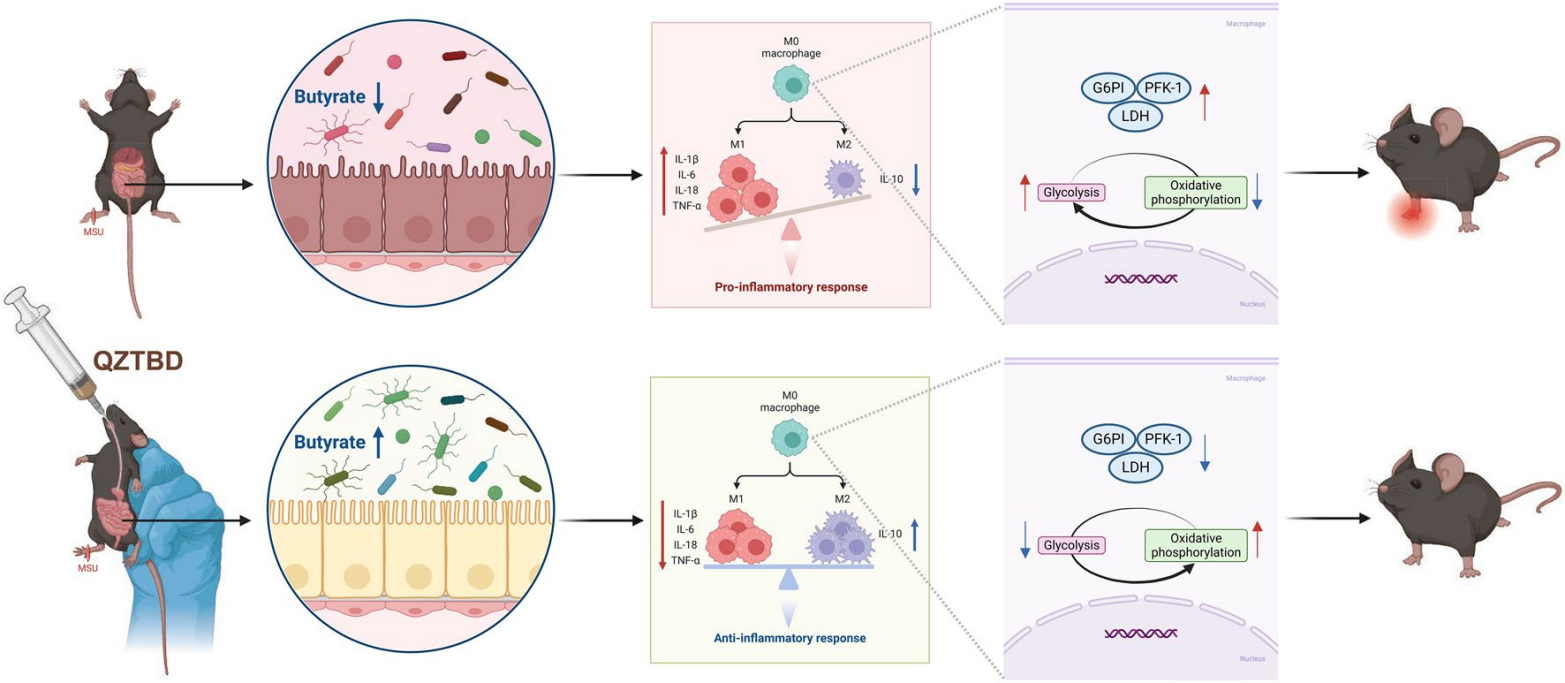
Mechanistic illustration of the QZTBD-mediated alleviation of gouty arthritis. The absence of butyrate-producing bacteria in *Uox*-KO mice results in a reduction of butyrate levels. Butyrate deficiency drives pro-inflammatory M1 macrophage polarization via glycolytic reprogramming, exacerbating the progression of gouty arthritis. QZTBD treatment restores gut microbial homeostasis, elevates butyrate production, and reprograms macrophage metabolism from glycolysis to OXPHOS. BPB and butyrate supplementation mimics the therapeutic effects of QZTBD, confirming the critical role of microbiota-derived butyrate in metabolic-immune crosstalk. Investigating the impact of QZTBD on bacterial butanoate metabolism represents a promising avenue for understanding its beneficial effects. Targeting the gut microbiota-butyrate-macrophage axis provides a novel therapeutic paradigm for the management of gouty arthritis

Our comprehensive study sheds light on the therapeutic potential of targeting macrophage metabolism via TCM-mediated gut microbiota modulation. These findings not only advance the mechanistic understanding of macrophage plasticity in gouty arthritis but also lay the groundwork for developing microbiota-centric therapeutic strategies against the inflammatory disorder. Specifically, QZTBD exerts its therapeutic effects through restoration of gut microbial homeostasis and butyrate production (Fig. 10), via butyrate-mediated metabolic reprogramming of macrophages. Future research should elucidate molecular mechanisms of butyrate-driven immunomodulation and explore the translational potential of microbiota-directed interventions in gouty arthritis management, including clinical trials to validate efficacy.

## Conclusion

Our research demonstrates that QZTBD alleviates gouty arthritis by restoring butyrate metabolism and modulating macrophage polarization. The results indicate that QZTBD increases the abundance of butyrate-producing bacteria, enhances butyrate levels, and thereby shifts macrophages from the pro-inflammatory M1 to the anti-inflammatory M2 phenotype. Glycolytic reprogramming in macrophages plays a crucial role in gout progression. Targeting macrophage polarization via probiotics and prebiotics offers a promising therapeutic strategy for gouty arthritis.

## Supplementary Information

Supplementary Material 1

Supplementary Material 2

## Data Availability

The raw sequencing data of this study have been deposited in the National Center of Biotechnology Information (NCBI) Sequence Read Archive (SRA) database under the BioProject accession number PRJNA1152246 and PRJNA896154. Additional data related to this paper may be requested from the authors.

## Abbreviations

ALT: Alanine aminotransferase
AST: Aspartate aminotransferase
BBR: Benzbromarone
BMDM: Bone marrow-derived macrophage
BPB: Butyric acid-producing bacteria
BUK: Butyrate kinase
BUT: Butyryl-CoA:acetate CoA-transferase
BUN: Blood urea nitrogen
CREA: Creatinine
ECAR: Extracellular acidification rate
FACS: Fluorescence-activated cell sorting
FMT: Fecal microbiota transplantation
GA: Gouty arthritis
GC-MS: Gas chromatography-mass spectrometry
HE: Hematoxylin and eosin
HUA: Hyperuricemia
IF: Immunofluorescence
IFN-γ: Interferon-γ
IL-1β: Interleukin-1β
IL-6: Interleukin-6
IL-10: Interleukin-10
IL-18: Interleukin-18
KEGG: Kyoto encyclopedia of genes and genomes
LPS: Lipopolysaccharide
LDH: Lactate dehydrogenase
M-CSF: Macrophage colony-stimulating factor
MSU: Monosodium urate
OCR: Oxygen consumption rate
PFK-1: 6-Phosphofructokinase-1
RT-qPCR: Reverse transcription quantitative polymerase chain reaction
SCFAs: Short-chain fatty acids
QZTBD: Qu-zhuo-tong-bi decoction
QZ-FMT: Fecal microbiota transplantation from QZTBD-treated mice
TNF-α: Tumor necrosis factor-α
TCM: Traditional Chinese Medicine
UA: Uric acid
UHPLC-MS/MS: Ultra-high performance liquid chromatography-tandem mass spectrometry
2-DG: 2-Deoxy-D-glucose
