# Supplementary material for: Qu-zhuo-tong-bi decoction exerts gouty arthritis therapy by skewing macrophage polarization through butanoate metabolism
Source: Chin Med. 2025 Jul 22;20:115. doi: 10.1186/s13020-025-01162-6 (PMC12281712; doi:10.1186/s13020-025-01162-6)
Supplement: Supplementary file 1 — Supplementary Material 1 [file 13020_2025_1162_MOESM1_ESM.docx]

**SUPPLEMENTARY DATA**

**Qu-Zhuo-Tong-Bi decoction exerts gouty arthritis therapy by skewing macrophage polarization through butanoate metabolism**

**Siyue Song^a, 1^, Xianghui Wen^a, 1^, Fusen Chen ^a, 1^, Jiatao Li^a^, Kaiyue Shi^a^, Yu Lou^a^, Anyi Xu^c^, Chengping Wen^a, b, *^, Tiejuan Shao^a,b, *^**

^a^ College of Basic Medical Sciences, Zhejiang Chinese Medical University, Hangzhou, 310053, China.

^b^ Center for Innovative Basic Research in Autoimmune Diseases in Medicine, Hangzhou, 310053, China

^c^ First Clinical Medical College, Zhejiang Chinese Medical University, Hangzhou, 310053, China

**^1^ These authors have contributed equally to this work.**

**^*^ Corresponding authors:** Prof. Chengping Wen, Email: wengcp@163.com

Or Prof. Tiejuan Shao, Email: tiejuanshao@zcmu.edu.cn

**Table S1. Herbal composition and dosage of QZTBD**

| **Chinese name** | **English name** | **Scientific name** | **Plant part** | **Weight (g)** | **Occupied percent** |
| --- | --- | --- | --- | --- | --- |
| Tufuling | Glabrous greenbrier | Smilax glabra Roxb | Rhizome | 60 | 28.57% |
| Bixie | Dioscoreae Hypoglaucae Rhizoma | Dioscorea spongiosa J. Q. Xi, M. Mizuno et W. L. Zhao | Rhizome | 30 | 14.29% |
| Yumixu | Stigma Maydis | Zea mays L | Style and stigma | 15 | 7.14% |
| Yiyiren | Coicis Semen | Coix lacryma-jobi L. var. ma-yuen (Rom.Caill.) Stapf | Seed | 30 | 14.29% |
| Xixiancao | Siegesbeckiae Herba | Siegesbeckia orientalis L | Dry aboveground part | 18 | 8.57% |
| Jianghuang | Curcumae Longae Rhizoma | Curcuma longa L | Rhizome | 12 | 5.71% |
| Sangjisheng | Talxilli Herba | Taxillus chinensis (DC.) Danser | Branch and leaf | 15 | 7.14% |
| Yanhusuo | Corydalis Rhizoma | Corydalis yanhusuo (Y. H. Chou and Chun C. Hsu) W. T. Wang ex Z. Y. Su and C. Y. Wu | Tuber | 18 | 8.57% |
| Foshou | Citri Sarcodactμlis Fructus | Citrus medica L. var. sarcodactylis Swingle | Dry fruit | 12 | 5.71% |

**Table S2. Primary antibodies used for IF analysis**

| Antibodies | Dilution | Source | Cat# |
| --- | --- | --- | --- |
| F4/80 | 10 μg/mL | Abcam | #90247 |
| iNOS | 1:100 | Abcam | #115819 |
| CD206 | 1:100 | Abcam | #64693 |

**Table S3. Primers for RT-qPCR analysis**

| Primers | Forward sequences | Reverse sequences |
| --- | --- | --- |
| *Buk* | TGCTGTWGTTGGWAGAGGYGGA | GCAACIGCYTTTTGATTTAATGCATGG |
| *But* | GCIGAICATTTCACITGGAAYWSITGGCAYATG | CCTGCCTTTGCAATRTCIACRAANGC |
| *Roseburia* | GCGGTRCGGCAAGTCTGA | CCTCCGACACTCTAGTMCGAC |
| *Ruminococcus* | GAGTGAAGTAGAGGTAAGCGGAATTC | GCCGTACTCCCCAGGTGG |
| *Butyricicoccus* | ACCTGAAGAATAAGCTCC | GATAACGCTTGCTCCCTACGT |
| *Eubacterium_xylanophilum*_group | AGAGTTTGATCCTGGCTC | TGCTGCCTCCCGTAGGAGT |
| *Faecalibacterium* | GGAGGAAGAAGGTCTTCGG | AATTCCGCCTACCTCTGCACT |
| *16S rRNA* | GCAGGCCTAACACATGCAAGTC | CTGCTGCCTCCCGTAGGAGT |

**Table S4. Primary antibodies used for western blotting analysis**

| Antibodies | Dilution | Source | Cat# |
| --- | --- | --- | --- |
| G6PI | 1:1000 | Abcam | #ab66340 |
| PFK-1 | 1:1000 | Proteintech | #55028-1-AP |
| LDH | 1:1000 | Abcam | #ab52488 |
| β-Actin | 1:1000 | CST | #4970 |

**Table S5. Antibodies used for flow cytometry analysis**

| Antibodies | Fluorophore | Source | Cat# |
| --- | --- | --- | --- |
| CD45 | BV605 | BD Pharmingen | #563053 |
| F4/80 | BV421 | BD Pharmingen | #565411 |
| CD86 | PE-Cy7 | BD Pharmingen | #560582 |
| CD163 | APC | Thermo Fisher Scientific | #17-1631-82 |
| Fixable Viability Stain 700 | APC700 | BD Pharmingen | #564997 |

**Table S6. Top 10 compounds identified from QZTBD by UHPLC-MS/MS**

| **PubChem CID** | **NameEN** | **CompositeScore** | **Formula** | **mzmed** | **rtmed** | **ppm** | **type** |
| --- | --- | --- | --- | --- | --- | --- | --- |
| 162946315 | Cyathisterol | 1 | C28H42O2 | 409.310964 | 816.306 | 0.08805831 | neg |
| 56776282 | (2R,3S,4S,5R,6S)-2-(hydroxymethyl)-6-[4-(hydroxymethyl)-1-propan-2-ylcyclohex-3-en-1-yl] oxyoxane-3,4,5-triol | 1 | C16H28O7 | 355.172177 | 156.686 | 0.49857273 | pos |
| 76900 | 2-Hydroxyadenine | 1 | C5H5N5O | 152.056678 | 38.1416 | 2.11487712 | pos |
| 80642 | 5-Hydroxymethyl-2-furancarboxylic acid | 1 | C6H6O4 | 143.03385 | 49.9684 | 1.04940985 | pos |
| 70627 | 5-Hydroxymaltol | 1 | C6H6O4 | 143.033811 | 89.3812 | 1.32368165 | pos |
| 5495613 | 5-Hydroxy-2,2-dimethyl-10-(2-methylbut-3-en-2-yl) pyrano[3,2-g] chromen-8-one | 1 | C19H20O4 | 313.143466 | 80.8755 | 1.48781891 | pos |
| 74819487 | Ingol | 1 | C20H30O6 | 367.208125 | 218.621 | 0.34173478 | pos |
| 936 | Nicotinamide | 1 | C6H6N2O | 123.055044 | 37.648 | 0.3556261 | pos |
| 53849849 | Schemb8357710 | 1 | C10H12O5 | 213.074632 | 28.2687 | 2.9648275 | pos |
| 7463 | p-Cymene | 1 | C10H14 | 135.116796 | 67.9505 | 1.51169457 | pos |

**Supplementary procedure for detailed UHPLC-MS/MS method for QZTBD component analysis**

Gradient conditions used for UHPLC-MS/MS analysis of QZTBD extract were as follows: 0–11 min, 15–75% B; 11–12 min, 75–98% B; 12–14 min, 98% B; 14–14.1 min, 98–15% B; 14.1–16 min, 15% B. The mobile phases were 0.1% formic acid in water (A) and 0.1% formic acid in acetonitrile (B). The injection volume was 5 μL, and the flow rate was 0.5 mL/min.

MS was conducted on a Q Exactive Focus (Thermo, USA) in ddMS^2^ mode. The following mass spectrometric conditions were used: positive and negative electrospray ionization (ESI) modes, with capillaries of +4.0 kV and −3.6 kV, respectively; sheath gas 45 arb, aux gas 15 arb, capillary temperature 400 °C, resolution 70,000 (MS) and 17,500 (MS/MS), NCE 15/30/45 eV. Data were processed using XCMS and compounds were identified with an in-house MS/MS database.

**Supplementary procedure for glycolysis stress test assay and mitochondrial stress test assay**

Prior to assays, BMDMs were seeded at 20,000 cells/well in Seahorse XFe96 cell culture microplates and incubated for 24 h with QZTBD-containing serum. For extracellular acidification rate (ECAR) measurement, cells were equilibrated for 1 h at 37°C in a CO_2_-free incubator using XF basal medium supplemented with 10 mM glucose on the measurement day. Sequential injections were then performed on a Seahorse XF96 analyzer: 10 mM glucose was added to establish the basal glycolysis, followed by 1.0 μM oligomycin to determine maximum glycolytic capacity, and finally 50 mM 2-deoxyglucose to confirm glycolytic origin of ECAR. For oxygen consumption rate (OCR) assessment, cells were similarly equilibrated for 1 h in XF basal minimal medium supplemented with 10 mM glucose, 1.0 mM pyruvate, and 2.0 mM L-glutamine. OCR was measured utilizing the Seahorse XF Cell Mito Stress Kit (Agilent) with sequential injections of 1.0 μM oligomycin, 1.0 μM FCCP, and 0.5 μM rotenone/antimycin A. All data were analyzed using Wave 2.6.1 software.

**Supplementary procedure for butyrate detection**

Butyrate was extracted from acidified fecal suspension and serum samples using anhydrous diethyl ether. 1 μL of processed samples or butyrate calibration standards were injected in split mode at a ratio of 5:1. A standard curve was established using a concentration series of butyrate standards. Helium carrier gas was maintained at a flow rate of 1.2 mL/min, with the inlet temperature set at 250°C and ion source temperature at 230°C. The initial column temperature was 100°C, ramped to 140°C at 6°C/min, and then to 220°C at 8°C/min. The data were acquired in Scan/SIM mode, with different groups of samples interspersed for analysis.

**

**

**Fig. S1.** (A) Significantly elevated S-UA levels in *Uox*-KO mice compared to WT controls. (B) Representative images and H&E-stained histology of footpads in *Uox*-KO mice (Scale bar: 100 μm). (C) Increased footpad swelling index and (D) reduced pain threshold in *Uox*-KO mice. n = 8 mice per group. Data are presented as mean ± SEM. ***P* < 0.01, ****P* < 0.001.

**
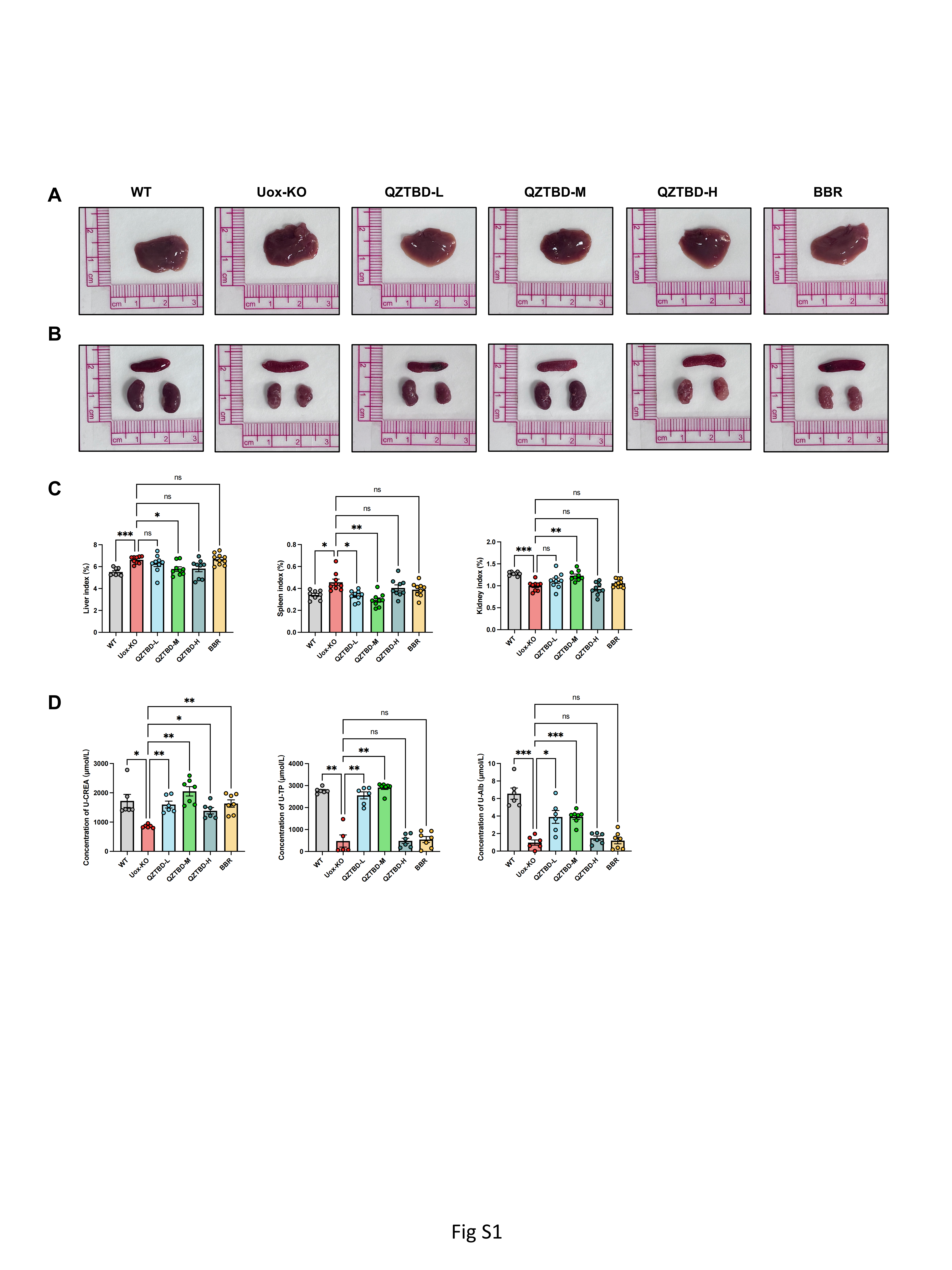
**

**Fig. S2.** (A-C) Pathological indications of liver, spleen, and kidney after QZTBD and BBR treatment in *Uox*-KO mice. (D) Indices of kidney damage in *Uox*-KO mice after treatment with different doses of QZTBD. n = 6 - 8 mice per group. Data are presented as mean ± SEM. ns, not significant; **P* < 0.05, ***P* < 0.01, ****P* < 0.001.

**Fig. S3.** (A-B) Footpad swelling index after BPB and butyrate treatment. (Scale bar: 500 μm). (C-D) Representative flow cytometry plots of M1 and M2 macrophage cells in intestinal lamina propria and spleen. (E-F) Immunofluorescence of M1 and M2 macrophages in footpad (Scale bar: 50 μm). n = 6 - 8 mice per group. Data are presented as mean ± SEM. ***P* < 0.01.

**
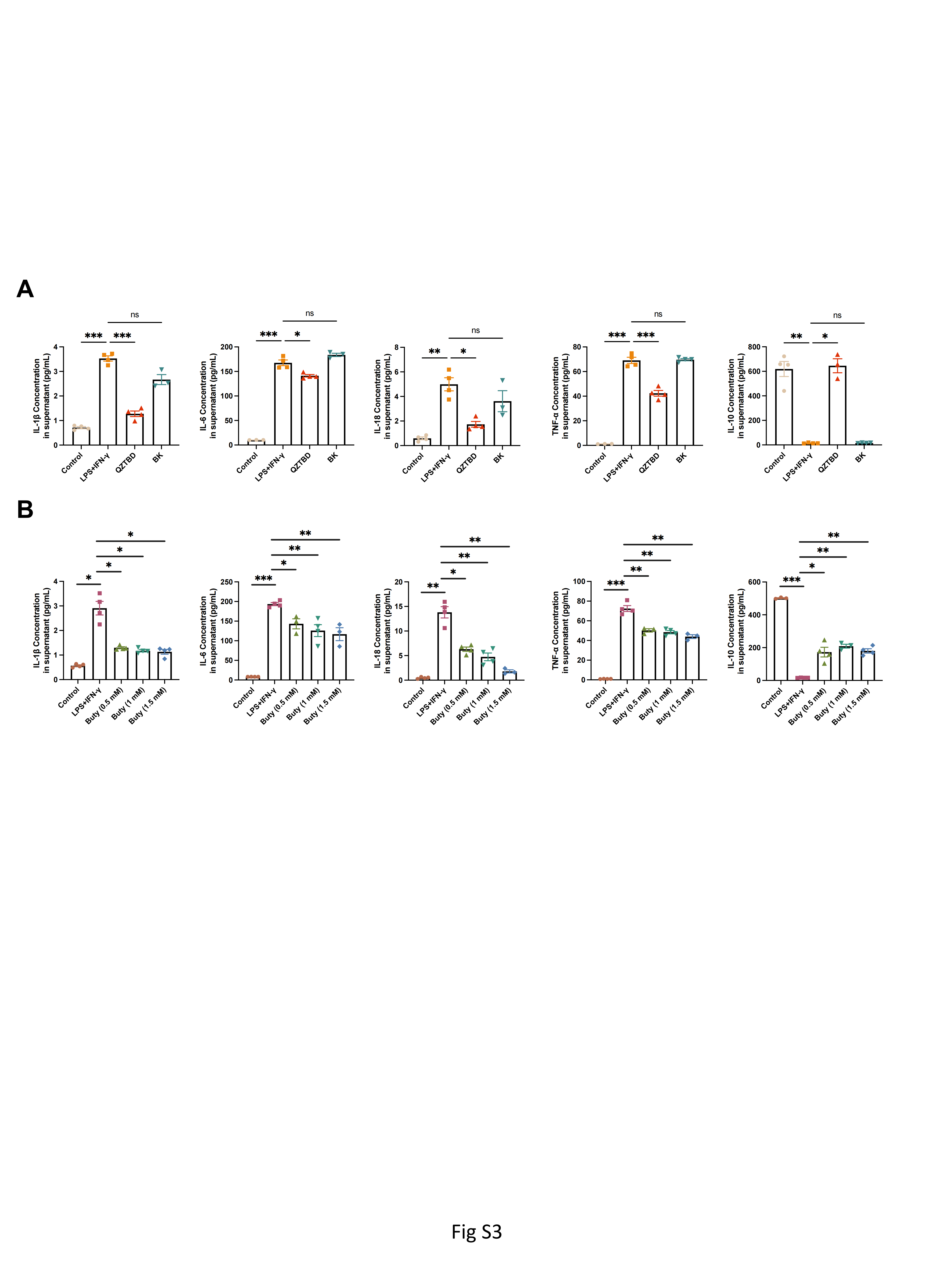
**

**Fig. S4.** (A-B) Cytokine levels (IL-1β, IL-6, TNF-α, IL-18, and IL-10) in BMDMs after butyrate intervention. n = 4 mice per group. Data are presented as mean ± SEM. ns, not significant; **P* < 0.05, ***P* < 0.01, ****P* < 0.001.

**

**

**Fig. S5.** (A) Changes of ECAR and OCR in BMDMs after 2-DG treatment. n= 5 mice per group. Data are presented as mean ± SEM. ***P* < 0.01, ****P* < 0.001.

**
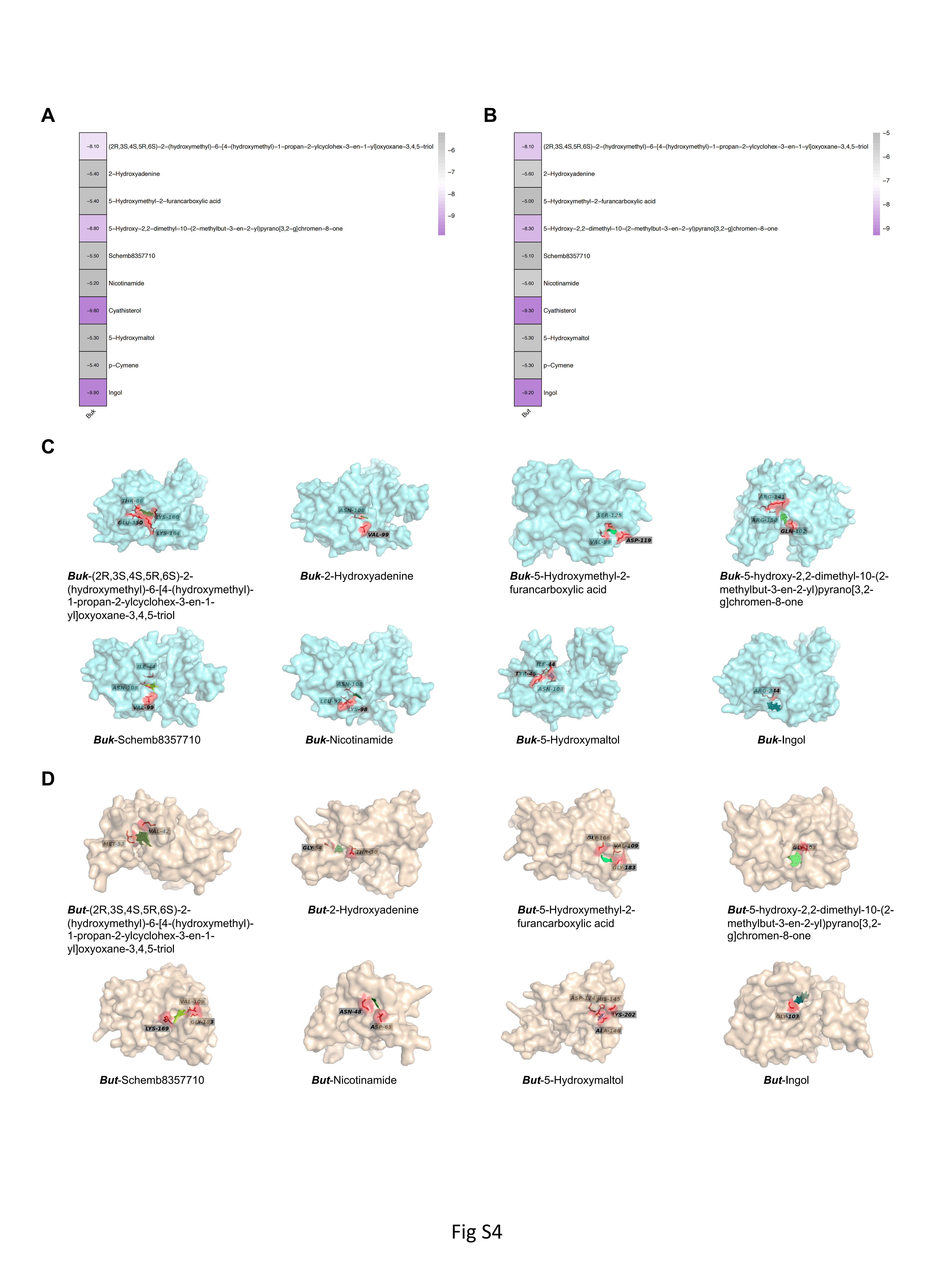
**

**Fig. S6.** (A-B) Heatmap of the binding energy values for the top 10 identified compounds from QZTBD against Buk and But targets. (C-D) Representative 3D binding diagram and schematic hydrogen bonding diagram of the main QZTBD components complexed with Buk and But. The image illustrates configurations of key components that formed hydrogen bonds in the binding pocket.

**
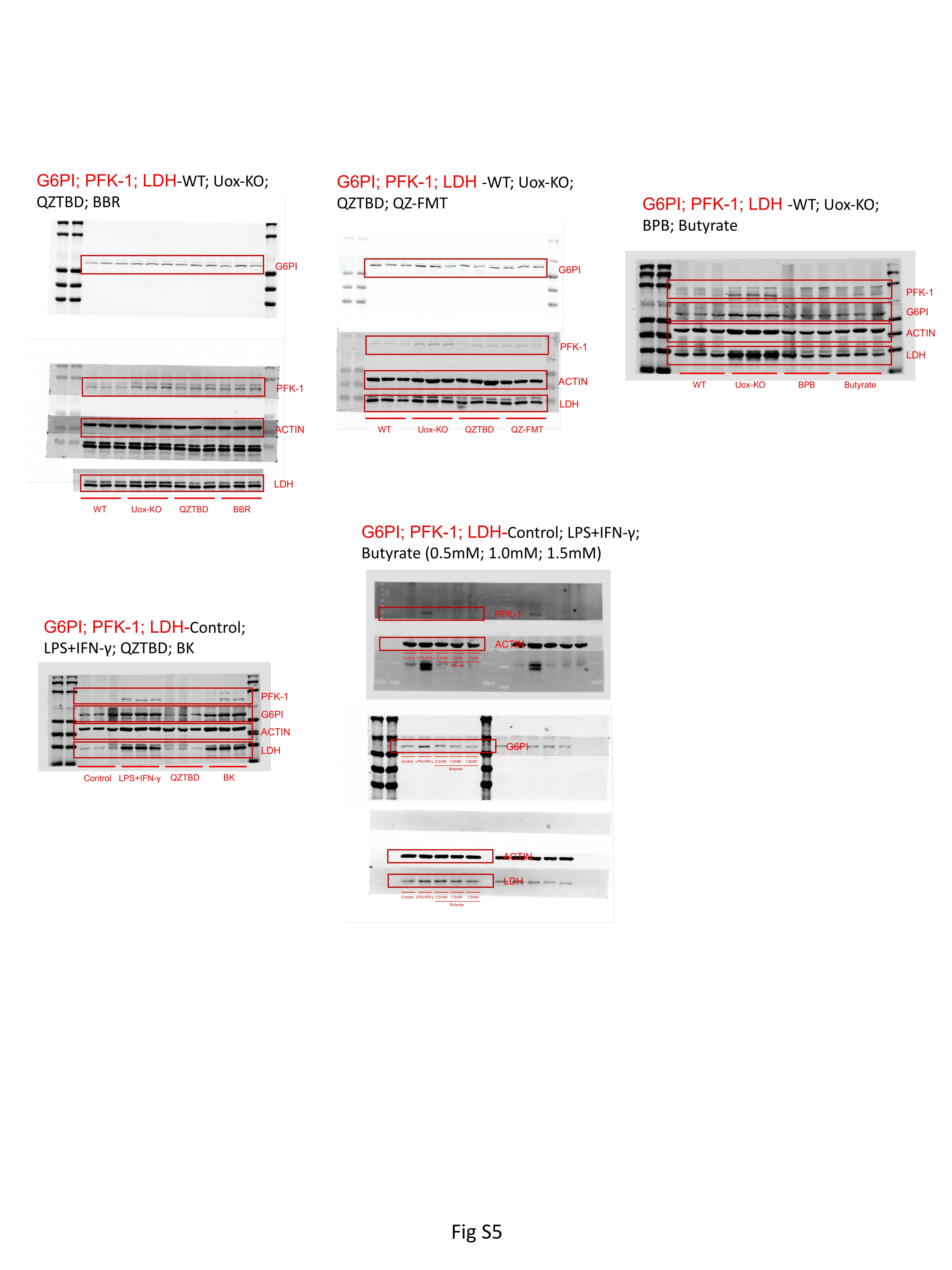
**

**Fig. S7.** The whole uncropped images of the original western blots.
